# Randomized phase II – study evaluating EGFR targeting therapy with Cetuximab in combination with radiotherapy and chemotherapy for patients with locally advanced pancreatic cancer – PARC: study protocol [ISRCTN56652283]

**DOI:** 10.1186/1471-2407-5-131

**Published:** 2005-10-11

**Authors:** R Krempien, MW Muenter, PE Huber, S Nill, H Friess, C Timke, B Didinger, P Buechler, S Heeger, KK Herfarth, A Abdollahi, MW Buchler, J Debus

**Affiliations:** 1 Department of Radiation Oncology, University of Heidelberg, Im Neuenheimer Feld 400, 69120 Heidelberg, Germany; 2 Department of Radiation Oncology, German Cancer Research Center (dkfz), 69120 Heidelberg, Germany; 3 Department of Surgery, University of Heidelberg, Im Neuenheimer Feld 110, 69120 Heidelberg, Germany; 4 Department of Medical Physics, German Cancer Research Center (dkfz), 69120 Heidelberg, Germany; 5 Merck KGaA, Frankfurter Str. 250, 64293 Darmstadt, Germany

## Abstract

**Background:**

Pancreatic cancer is the fourth commonest cause of death from cancer in men and women. Advantages in surgical techniques, radiation therapy techniques, chemotherapeutic regimes, and different combined-modality approaches have yielded only a modest impact on the prognosis of patients with pancreatic cancer. Thus there is clearly a need for additional strategies. One approach involves using the identification of a number of molecular targets that may be responsible for the resistance of cancer cells to radiation or to other cytotoxic agents. As such, these molecular determinants may serve as targets for augmentation of the radiotherapy or chemotherapy response. Of these, the epidermal growth factor receptor (EGFR) has been a molecular target of considerable interest and investigation, and there has been a tremendous surge of interest in pursuing targeted therapy of cancers via inhibition of the EGFR.

**Methods/design:**

The PARC study is designed as an open, controlled, prospective, randomized phase II trial. Patients in study arm A will be treated with chemoradiation using intensity modulated radiation therapy (IMRT) combined with gemcitabine and simultaneous cetuximab infusions. After chemoradiation the patients receive gemcitabine infusions weekly over 4 weeks. Patients in study arm B will be treated with chemoradiation using intensity modulated radiation therapy (IMRT) combined with gemcitabine and simultaneous cetuximab infusions. After chemoradiation the patients receive gemcitabine weekly over 4 weeks and cetuximab infusions over 12 weeks. A total of 66 patients with locally advanced adenocarcinoma of the pancreas will be enrolled. An interim analysis for patient safety reasons will be done one year after start of recruitment. Evaluation of the primary endpoint will be performed two years after the last patient's enrolment.

**Discussion:**

The primary objective of this study is to evaluate the feasibility and the toxicity profile of trimodal therapy in pancreatic adenocarcinoma with chemoradiation therapy with gemcitabine and intensity modulated radiation therapy (IMRT) and EGFR-targeted therapy using cetuximab and to compare between two different methods of cetuximab treatment schedules (concomitant versus concomitant and sequential cetuximab treatment).

Secondary objectives are to determine the role and the mechanism of cetuximab in patient's chemoradiation regimen, the response rate, the potential of this combined modality treatment to concert locally advanced lesions to potentially resectable lesions, the time to progression interval and the quality of life.

## Background

Pancreatic cancer is the fourth commonest cause of death from cancer in men and women [1,2]. Surgical therapy currently offers the only potential monomodal cure for pancreatic adenocarcinoma [3]. However only a few patients present with tumors that are amenable to resection, end even after resection of localized cancers, long term survival is poor. At presentation, only 20% of patients with pancreatic adenocarcinoma have resectable cancers, 40% have locally advanced tumors, and 40% have metastatic disease [5].

However, long-term (5-year) survival rates – even for patients undergoing "complete" resection – are below 20% [4,5]. Loco-regional recurrence and/or metastatic disease develop in the majority of patients who undergo pancreatic resection. Relapse occurs within 9–15 months after initial presentation and patients have median life expectancies of only 12–15 months without adjuvant therapy [4]. The 5-year survival rate of patients with resected pancreatic adenocarinoma is approximately 10% [6]. The statistics for the 80 to 90 % of patients who present with locally advanced and metastatic pancreatic cancer are even more dismal. Rarely do such patients achieve a complete response to treatment; median survival is 5–10 months and 5-year survival is near zero [7].

Both distant and local/regional patterns of recurrence are common, and this suggests that most patients have occult metastatic disease or local/regional (or both) at the time of resection. Postoperative chemoradiationtherapy (CRT) has been shown to improve survival in patients with resected pancreatic adenocarcinoma [8-10], although there is debate over whether radiotherapy is a beneficial component [5,11]. The problems with the postoperative adjuvant approach include the fact that at least 25% of patients do not actually receive adjuvant therapy because of complications of surgery or patient refusal [10,12]. A primary advantage of preoperative therapy is therefore the assurance that CRT is received by all patients in a timely fashion. Other benefits are the delivers of radiation to well-oxygenated tissues and the avoidance of radiation to fixed loops of intestine within the operative field. Another rationale for neoadjuvant treatment is that occult metastatic disease is given the opportunity to manifest, thus allowing patients to avoid the morbidity of resection or laparotomy. Finally, the potential for preoperative CRT to convert locally advanced lesions to resectable lesions could greatly increase the number of patients with pancreatic cancer who might be offered a chance of cure. Several trials could show that dose escalation in radiation therapy using either EBRT [8] or IORT [13,14] resulted in improved local control in combination with potentially curative resection. The efficacy of external beam irradiation (EBRT) in pancreatic cancer is limited by the inability to deliver adequate doses of irradiation secondary to the dose tolerance limits of small bowel, spinal cord, stomach, kidney, and liver [15]. Further, the use of combined modality approaches in pancreatic cancer is associated with increased gastrointestinal toxicity [16]. Technical developments like intensity-modulated radiation therapy (IMRT) have the potential to significantly improve radiation therapy of pancreatic cancers by reducing normal tissue dose, and simultaneously allow escalation of dose to further enhance locoregional control [17].

To achieve long-term success in treating this disease it is therefore increasingly important to identify effective neoadjuvant/adjuvant multimodality therapies.

Concurrent chemoradiation is the standard of care for locally advanced non metastatic pancreatic cancers. Median survival rates vary between different trials depending on their selection criteria between 7 and 12 month, while 1 year overall survival is between 30% and 45% [18].

Systemic chemotherapy, a mainstay of pancreatic cancer treatment, essentially has been ineffective until recently when gemcitabine became available [19]. In a phase III trial comparing gemcitabine versus 5-flurouracil in advanced pancreatic cancer, patients who received gemcitabine showed a modest improvement in response rate, a marginal survival advantage, and most important, superior clinical response [20]. Therefore gemcitabine became the standard treatment of advanced pancreatic cancer. Despite these results, however median survival duration in patients with advanced pancreatic cancer continues to be less than 6 month.

Still, advantages in surgical techniques, radiation therapy techniques, chemotherapeutic regimes, and different combined-modality approaches have yielded only a modest impact on the prognosis of patients with pancreatic cancer. Thus there is clearly a need for additional strategies. One approach involves using the identification of a number of molecular targets that may be responsible for the resistance of cancer cells to radiation or to other cytotoxic agents. As such, these molecular determinants may serve as targets for augmentation of the radiotherapy or chemotherapy response. Of these, the epidermal growth factor receptor (EGFR) has been a molecular target of considerable interest and investigation, and there has been a tremendous surge of interest in pursuing targeted therapy of cancers via inhibition of the EGFR [21,22].

The overexpression of EGFR has been demonstrated in a number of human tumor types, including head-and-neck cancers, colon cancer, breast cancer, gliomas, lung cancer and pancreatic cancer [23,24]. The rationale for investigating of EGFR inhibitors as radiation sensitizers in cancer therapy is based on the following observations: (1) positive correlation between EGFR expression and cellular resistance to radiation in many cell types [23]; (2) the degree of radioresistance correlates positively with the magnitude of EGFR overexpression [25]; (3) cell survival and repopulation during a course of radiotherapy are influenced by activation of EGFR/transforming growth factor alpha that is induced after exposure to radiation [26]; and (4) inhibition of EGFR signaling-enhanced radiation sensitivity [27,28].

Cetuximab is a monoclonal antibody that specifically binds to the EGFR, thereby inhibiting downstream signal transduction pathways [29]. It has been shown in vivo and in vitro to enhance radiosensitivity, to promote radiation induced apoptosis, to decrease cell proliferation, to inhibit radiation-induced damage repair, and to inhibit tumor angiogenesis [23].

Phase I studies have shown that cetuximab has tolerable toxic effects. Acneiform rash is the most common toxic effect. Besides skin toxicity cetuximab has the potential to cause allergic reactions, including anaphylaxis. However, this has not been shown to be a significant clinical problem [23].

Phase I and II clinical studies on EGFR antibodies given as a single agent were performed in patients with advanced NSCLC, ovarian, head and neck, prostate, and colorectal cancer. Stable disease with tolerable side effects was seen in about 20% of the patients [23]. Several phase I-III trials testing the effect of different EGFR inhibitors combined with radiotherapy or radiochemotherapy are currently ongoing. The so far most important trial testing EGFR inhibition in combination with radiotherapy was a randomized phase III trial testing radiotherapy alone versus radiotherapy plus cetuximab [30]. 424 patients with loco-regionally advanced squamous cell carcinoma or head-and-neck cancers were randomized into curative radiotherapy plus/minus cetuximab. Cetuximab was applied once at a dose of 400 mg/m^2 ^in the week prior to radiotherapy (week1) and weekly during the course of radiotherapy at a dose of 250 mg/m^2 ^before irradiation. Locoregional tumor control rates after 1 and 2 years were 69% and 56% for patients treated simultaneously with cetuximab versus 59% and 48% for patients who received radiotherapy alone (p = 0.02). Overall survival rates at 2 and 3 years after treatment were 62% and 57% for cetuximab treated patients and 55% and 44% for patients with irradiation alone (p = 0.02). Median survival times were 54 months (95%C.I.36;58) and 28 months (21;38), respectively.

A recently completed phase II trial of cetuximab in combination with gemcitabine for patients with advanced pancreatic cancer showed promising results [31]. In that trial 41 patients received cetuximab with gemcitabine and were evaluated for efficacy and toxicity. The toxicity profile of this combination was consistent with that of gemcitabine, except for acneiforme rash induced by cetuximab. A partial response rate of 12.2% (5 patients) and stable disease rate of 63.4% was observed in this study. Medial time to progression and medial survival duration were 3.8 months and 7.1 months, respectively, while 1-year survival rates and progression-free survival rates were 31.7% and 12%, respectively. This 1-year survival is considerably better than that achieved using gemcitabine alone as documented in a previous phase III trial [20]. Recently a phase three study could demonstrate the benefit of the combination of an EGFR tyrosine kinase inhibitor in combination with chemotherapy in pancreatic cancer [32]. A total of 569 patients with advanced pancreatic cancer were randomized to receive standard dose gemcitabine, 1000 mg/m2 iv weekly in 7 out of 8 weeks, than weekly 3 out of four weeks plus either erlotininb 100 mg daily (n = 285) or placebo (n = 284). Combined erlotinib therapy with gemcintabine resulted in a 24% improvment in survival as compared to placebo (p = 0.025) with corresponding 1-year survival rate of 24% and 17% (erlotinib and placebo arm, respectively).

Regarding the benefits of EGFR-targeted therapy in the combined modality treatment using either irradiation or chemotherapy, it is increasingly becoming clear that EGFR-targeted therapy is an important novel strategy for the treatment of pancreatic cancers. Since chemoradiation is the standard of care for locally advanced non metastatic pancreatic cancer, there has been considerable interest in gaining increased experience with this therapy in combination with chemoradiation in an effort to evaluate the efficacy and the toxicity profile of this regimen.

## Methods/design

### Trial organization

PARC has been designed by the Trial Center of the Department of Radiation Oncology, University of Heidelberg. The trial is carried out by the Department of Radiation Oncology together with the German Cancer Research Center (DKFZ) and Department of Surgery. The trial is an investigator initiated trial. Trial medication (cetuximab) is supplied by Merck KGaA, Darmstadt, Germany.

### Coordination

The trial is co-ordinated by the Department of Radiation Oncology in cooperation with the DKFZ and the Department of Surgery at the University of Heidelberg. The Dept. of Radiation Oncology is responsible for overall trial management, trial registration (International Standard Randomized Controlled Trial Number [ISRCTN 56652283], ), database management, quality assurance including monitoring, reporting and for the scientific program of all trial related meetings).

### Investigators

Patients will be recruited by the Department of Radiation Oncology at the University of Heidelberg. Due to the multi-modal nature of the trial, all investigators are experienced oncologists from the fields of radiation oncology, hematology/oncology, and general surgery at the University of Heidelberg co-operating in this trial.

### Adverse events committee

This committee consists of 3 independent physicians (medical oncologist, radiation oncologist and surgeon) and decides on the final diagnostic classification of critical clinical events. For all serious adverse events the documentation and relevant patient data are verified by the co-ordinating personnel before submitting the data to the Adverse Events Committee for diagnostic classification.

Analysis of safety related data is performed with respect to frequency of:

• Serious Adverse Events and Adverse Events stratified by organ-system

• Adverse Events stratified by severity

• Adverse Events stratified by causality.

Patient toxicities will be assessed using the NCI Common Toxicity Criteria (CTC). Toxicity will be evaluated pretreatment, weekly during chemoradiation /chemotherapy, prior to each course of infusional Cetuximab and at follow-up. Unacceptable toxicity is defined as unpredictable, or irreversible Grade 4 toxicity. Decisions regarding weekly chemoradiation treatment, chemotherapy dose-adjustment, and cetuximab dose-adjustment will be made using the guidelines below and based on hematological parameters (ANC and platelets) monitored weekly during chemoradiation before each dose of cetuximab and gemcitabine.

### Medication supply

All chemotherapeutic agents are prepared and provided by the pharmacy of the University Hospital Heidelberg. Cetuximab is provided by Merck KGaA, Darmstadt, and is stored by the pharmacy of the University Hospital Heidelberg. Medication will be prepared for each patient specifically and delivered just prior to administration to the Department of Radiation Oncology.

### On-site monitoring

During recruitment of patients monitoring on site is performed according to good clinical practice (GCP) guidelines. The data management will be performed by the Trial Center of the Department of Radiation Oncology, University of Heidelberg. The medical monitoring will be done by two independent oncologists not involved in conducting this trial.

### Ethics, informed consent and safety

The final protocol was approved by the ethics committee of the University of Heidelberg, Medical School (L-283/2004,  Paul-Ehrlich-Institute (PEI) registration number 1205/01). This study complies with the Helsinki Declaration in its recent German version, the Medical Association's professional code of conduct, the principles of Good Clinical Practice (GCP) guidelines and the Federal Data Protection Act. The trial will also be carried out in keeping with local legal and regulatory requirements. The medical secrecy and the Federal Data Protection Act will be followed.

Written informed consent is obtained from each patient in oral and written form before inclusion in the trial and the nature, scope, and possible consequences of the trial have been explained by a physician. The investigator will not undertake any measures specifically required only for the clinical trial until valid consent has been obtained.

### Patient selection

PARC focuses on hospitalized patients over 18 years of age treated with pancreatic head resection for pancreatic adenocarcinoma during an 18-months period started in August 2004. Men and women over eighteen years of age with locally advanced pancreatic adenocarcinoma will be screened for participation in the study. A detailed overview of all eligibility criteria is given in Table 2.

**Table 2:** Eligibility Criteria

**Inclusion criteria**	**Exclusion criteria**
• Age equal or greater than 18 years • Primary inoperable locally advanced pancreatic adenocarcinoma • No evidence of metastatic disease. • Hb >10.0 g/%, WBC >3,000 cells/mm^3^, platelets >100,000 cells/mm^3^. • Performance status: Karnofsky ≥70. • No acute infections at the time of therapy initiation. • Patient must be able to give informed consent • Patient has given informed consent	• Active infection • Liver function impairment • Pregnancy or breastfeeding. • Metastatic disease • Other severe systemic disease • Second malignancy (except carcinoma in situ of the cervix uteri, basal cell carcinoma of the skin after adequate oncologic treatment) • Any other experimental treatment 4 weeks before study inclusion • Known positive HACA (human antichimeric antibody) • Known allergy against extrinsical proteins • Previous antibody therapy • Allergy against iv contrast agent (for CT-scans) • Previous chemo- and/or radiation treatment or EGFR-inhibitor therapy for pancreatic cancer • Lack of compliance • Inability to follow the instructions given by the investigator or the telephone interviewer (insufficient command of language, dementia, lack of time) • Lack of informed consent

### Study design

The PARC study is designed as an open, controlled, prospective, randomized feasibility phase II trial meant to evaluate the efficacy and toxicity of chemoradiation in combination with cetuximab for patients with locally advanced pancreatic cancer. Compared are chemoradiation with simultaneous cetuximab versus chemoradiation with simultaneous/sequential cetuximab. The treatment is offered to a heterogeneous group of people under clinical circumstances, covering a wide age range, for both sexes and with heterogeneous characteristics / co-morbidities.

One year after inclusion of the first patient an interim analysis will be performed. The study design will not be changed prior to agreement of the ethics committee.

### Study objectives

The primary objective is to evaluate the feasibility and the toxicity profile of this regimen and to compare between two different methods of cetuximab treatment schedules (concomitant versus concomitant and sequential cetuximab treatment) in combination with chemoradiation therapy with gemcitabine and intensity modulated radiation therapy.

Secondary objectives are to determine the role and the mechanism of cetuximab in patient's chemoradiation regimen, the response rate, the potential of this combined modality treatment to concert locally advanced lesions to potentially resectable lesions, the time to progression interval and the quality of life.

### Randomization and standardized treatment scheme

A block-randomization-list is generated via computer system (SAS Version 8.2, SAS Institute Inc., Cary, USA). The sealed randomization list is stored in the investigator file. Patients are randomized using sealed opaque envelopes in the independent study center at the Department of radiation oncology until informed consent is attained and diagnostic procedures rule out any contra-indication for participation in this trial.

After randomization and pre-treatment evaluation treatment must begin within 2 weeks.

All patients will receive a combination with radiotherapy, gemcitabine weekly and cetuximab weekly.

#### Study arm A

Cetuximab will be given as loading dose 400 mg/m^2 ^over 120 minutes on day 1. On day 8,15,22,29, 36 (5 doses) cetuximab 250 mg/m^2 ^over 60 minutes will be given simultaneously with radiation. Non-steroidal anti-inflammatory drugs and steroids will be given before cetuximab.

Gemcitabine 300 mg/m^2 ^over 60 minutes will be given on day 12,19,26,33,40 (5 doses) 2 hours after radiation therapy. Sequential chemotherapy with gemcitabine weekly 1000 mg/m^2 ^over 60 minutes will be continued after finishing radiotherapy on day 47, 54, 62. The timing of these courses will be adjusted in patients who have treatment interruptions.

External beam radiation is to be given concurrently with chemotherapy and cetuximab with a total dose of 54 Gy in 25 fractions over 5 weeks. Patients are to be treated using an integrated intensity modulated radiation therapy (IMRT) boost concept, which allows the use of different single doses for the gross target volume (GTV) and the clinical target volume (CTV) in one fraction. GTV includes only the gross tumor volume, whereas CTV includes the primary tumor and the regional lymphnodes including the hepatoduodenal ligament, origins of the celiac axis and superior mesenteric artery. The median total dose for the GTV is to be 54.0 Gy (single dose 2.16 Gy) and for the CTV 45.0 Gy (single dose 1.8 Gy). The dose constraints for stomach, duodenum, small intestine, colon are 45 Gy in the maximum, mean dose for kidneys should be below 10 Gy, only one third of the kidneys should receive more than 20 Gy. KonRad™ (Siemens Oncology Systems, Concorde, USA) will be used for inverse treatment planning. Treatment will be performed using step-and-shoot IMRT and stereotactic target point localization with 7 coplanar fields and 50 to 65 segments. Average treatment time will be 10 minutes. Patients are to be fixed during therapy by individual immobilization devices.

#### Study arm B

Cetuximab will be given as loading dose 400 mg/ m^2 ^over 60 minutes on day 1. On day 8,15,22,29, 36 (5 doses) cetuximab 250 mg/m^2 ^over 60 minutes will be given simultaneously with radiation. Non-steroidal anti-inflammatory drugs and steroids will be given before cetuximab.

Sequential cetuximab 250 mg/ m^2 ^over 60 minutes will be given weekly beginning on day 46, over 3 month (12 doses)

Gemcitabine will be given as in study arm A.

External beam radiation will be given as in study arm A.

Restaging using computed tomography will be performed 5 weeks after completion of radiotherapy and at the end of sequential cetuximab administration.

**Figure 1:**
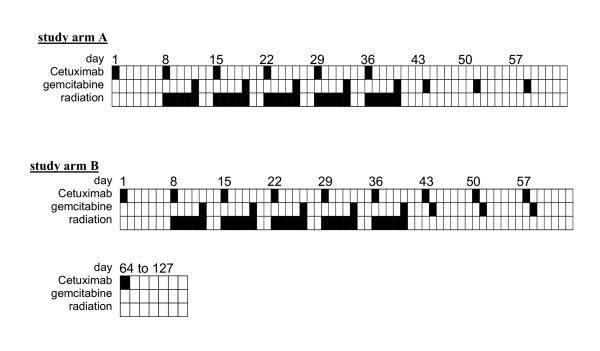
PARC treatment scheme.

The treatment protocol is outlined in figure 1.

### Investigation schedule and follow-up

All patients (study arm A or B) must have appropriate lab and radiographic studies (CXR; bone scintigraphy, abdominal ultrasound, CT abdomen; CBC; platelet count; BUN; creatinine; bilirubin, CA 19-9, and CEA) conducted prior to study enrolment to meet eligibility criteria.

During days 1–70 [study arm A] or days 1–130 [study arm B] patients will be assessed with laboratory evaluation: complete blood count and blood chemistries weekly. Laboratory parameters in both study arms will be evaluated before each dose of gemcitabine, in study arm B during cetuximab weekly before cetuximab.

Vital signs (blood pressure and pulse rate) and temperature are controlled daily during treatment. Patients are evaluated prior to receiving chemoradiation or chemotherapy. Patients enrolled in study arm A and B are evaluated weekly by the radiation oncology team during treatment. The team will check patients at each visit for symptoms due to therapy; a physical examination and complete safety labs should be performed. The quality of life questionnaire will be filled out during weeks 1, 9 and 17.

During post-chemoradiation, infusional cetuximab weekly (Study Arm B) patients will be evaluated by a physician prior to treatment and every 2 to 3 weeks with clinical assessment and laboratory parameters including a CBC, electrolytes, BUN, and creatinine.

In the post-treatment period patients will be seen every 3 months by the radiation oncology department for the first 2 years, every 4 months for the third year, and every 6 months during the 4th and 5th post-treatment years.

The aggregate clinical, laboratory, and imaging evaluations required per protocol as well as the timing of the optional quality of life questionnaire are outlined in table 1.

**Table 1:** 

**Investigation**	**Pretreatment**	**Chemoradiation / Chemotherapy weekly**	**Each Course of Infusional Gemcitabine/ Cetuximab**	**follow-up**
**Clinical examination**	X	X	X	X
**Laboratory tests**	X^a^	X	X	X
**Tumor markers**	X^b^	X	X	X
**CT Abdomen**	X			X^*c*^
**CXR, PA, LAT**	X		X	X
**Renal function**	X^*d*^			
**Pregnancy Test**^f^	X			
**SAE**	X	X	X	
**QOL Survey**	X^*e*^	X	X	X

a. Includes Electrolytes, BUN, Creatinine, SGOT, Alk Phos, Total Bilirubin, Albumin, Glucose, Calcium, auto-antibodies

b. Includes CEA and CA 19-9

c. A CT scan will be performed 4 weeks after chemoradiation and than every 3 months.

d. Includes CT with i.v. contrast

e. QLQ-C30, QLQ-Pan 26

f. If indicated

The follow-up will be continued for two years. Follow-up data of overall survival will be evaluated annually.

### Assessment of quality of life

Measurement of quality of life is one of the secondary objectives of the trial. Overall survival, return to previous employment as well as persistence of symptoms, the ability to perform appropriate activities and to care for oneself are criteria applied in the three questionnaires used in this study.

EORTC QLQ-C30 is a general measure of qualitiy of life in cancer patients. It incorporates nine multi-item scales: five functional scales (physical, role, cognitive, emotional, and social); three symptom scales (fatigue, pain, and nausea and vomiting); and a global health and quality-of-life scale [33]. Specific symptoms (dyspnoea, insomnia, anorexia, constipation, diarrhoea, and financial impact) are measured as six single items. This instrument has been used extensively with a variety of cancer patients and was able to discriminate between individuals with metastatic and non-metastatic disease, as well as between patients at different stages of illness. The scale has good internal consistency (alpha > 0.70), and good test re-test reliability (0.80 to 0.90) [34].

To assess disease-specific symptoms for patients with pancreatic cancer the pancreatic specific module (QLQ-PAN26) [35] that has been designed to use long with the general measure is used in this study.

### Evaluation of the role of cetuximab

An investigation of the effects and mechanism of cetuximab will be performed. Cetuximab has been shown in vivo and in vitro to enhance radiosensitivity, to promote radiation induced apoptosis, to decrease cell proliferation, to inhibit radiation-induced damage repair, and to inhibit tumor angiogenesis [23,24,36]. In view of the encouraging results achieved by using cetuximab in combination with other antineoplastic therapies, studies are now needed to define the molecular and immunologic mechanism(s) of this modality [30,31]. If the mechanism of action of cetuximab is more clearly understood it can be applied more selectively and its therapeutic index will be enhanced. Treatment related primary and acquired chemo- radioresistance presents a significant hindrance for all current therapy regimes in pancreatic cancer patients [23,24]. Multiple factors such as genetic instability of tumors and high inter- and intratumoral heterogeneity contributes to the hardly predictable therapy resistance [22]. To understand patterns of therapy response genome expression profiling and detection of genetic polymorphisms enables to identify key mechanisms in systems biology. Microarray technology will be used to identify predictors for therapy response or failure. The objectives are to correlate and potentially predict therapy response to cetuximab in combination with gemcitabine and radiotherapy using tumor genomic fingerprints. Tissue will be obtained either prior to neoadjuvant therapy by biopsy or during surgery. In order to perform the genomic approach patients biopsies are correlated and RNA and DNA isolation will be performed. After expression profiling the most promising differentially expressed genes are validated using real-time quantitative polymerase chain reaction. To predict the efficacy of neoadjuvant trimodal therapy additionally patients blood is collected before, during, and after neoadjuvant therapy to detect and correlate well known tumor and angiogenesis marker (VEGF, bFGF, IL8 etc.) using antibody chips.

### Statistical considerations and sample size estimation

The primary endpoint in this study is the feasibility and safety of the trimodal combination therapy with gemcitabine based chemoradiation and cetuximab. Secondary endpoints are overall survival period, measured from the date of therapy start. The one-year survival rates after chemoradiation with gemcitabine is 42 % [20,37]. The sample size calculation is based on the assumption of an increase of one-year survival rates up to 67% due to the triple therapy [30-32].

Assuming an accrual period of 24 months and a follow-up of 42 months, testing for a difference in hazard (hazard ratio ≠ 1) on level α = 0.05 and with a power of 80% a study sample size of 58 patients (29 patients per study arm) is needed. Taking into consideration the estimate of approximately 15% of patients which will not complete the treatment, a total number of 66 patients should be randomized.

The overall survival between both therapy arms will be compared using the Chi-square test. The overall survival will be summarized by Kaplan-Meier estimate and differences in therapy protocols will be analyzed by univariate Cox-regression.

Various secondary endpoints will be evaluated in this study as well, time to progression, measured from date of therapy start, will be summarized by Kaplan-Meier estimate. Further tumor response after 3, and 6 months and secondary operability will be calculated

One year after inclusion of the first patient an interim analysis will be carried out. The main evaluation will be performed two years after the last patient's enrolment.

There will be explicit stopping rules in place to terminate the trial early in the unlikely event that an unacceptably high rate of treatment related deaths (TRD) is observed. TRD will be monitored using the design of Thall and Simon [38]. A non-informative Beta prior distribution (i.e., B (0.015, 0.085) for TRD rate is assumed. The trial will be stopped if at any point during the trial there is a greater than 90% probability that the true TRD rate is greater than 0.05. Each patient will subsequently be evaluated and, an independent safety board will be consulted in making decision.

In view of the poor prognosis of the patient group, there will be no explicit stopping rules based on the overall number of toxicities, since even high rates of reversible toxicities seem acceptable if there is a large survival gain. Patients can withdraw from study participation at any time. Patients are taken off the study if unacceptable toxicity appears. Unacceptable toxicity is defined as unexpected serious side effects or irreversible Grade 4 toxicity. Patients who withdraw from the study may be treated with 5-FU and folinic acid or with gemcitabine. The decision will be based on the individual reasons for withdrawing from the study.

## Discussion

About 20–40% of patients present with a locally advanced pancreatic cancer which is not curable by resection [3,4]. The aim of primary chemoradiation in this situation is to achieve a local response with the aim prolonged survival and of preventing local tumor complications. Further downstaging or downsizing may enable secondary respectability. Chemoradiation in locally advanced pancreatic cancer results in significantly prolonged survival with 1-year survival about 40% compared to chemotherapy alone with 1-year survival about 20% [37]. Long-term survival is poor, rarely do such patients achieve a complete response to treatment; median survival is 5–10 months and 5-year survival is near zero [7].

Advantages in surgical techniques, radiation therapy techniques, chemotherapeutic regimes, and different combined-modality approaches have yielded only a modest impact on the prognosis of patients with pancreatic cancer [4]. Thus there is clearly a need for additional strategies. One approach involves using the identification of a number of molecular targets that may be responsible for the resistance of cancer cells to radiation or to other cytotoxic agents. As such, these molecular determinants may serve as targets for augmentation of the radiotherapy or chemotherapy response. Of these, the epidermal growth factor receptor (EGFR) has been a molecular target of considerable interest and investigation, and there has been a tremendous surge of interest in pursuing targeted therapy of cancers via inhibition of the EGFR [23,36].

Regarding the benefits of EGFR-targeted therapy in the combined modality treatment using either irradiation or chemotherapy, it is increasingly becoming clear that EGFR-targeted therapy is an important novel strategy for the treatment of pancreatic cancers [30-32]. Since chemoradiation is the standard of care for locally advanced non metastatic pancreatic cancer, there has been considerable interest in gaining increased experience with this therapy in combination with chemoradiation in an effort to evaluate the efficacy and the toxicity profile of this regimen.

Being a center focusing on pancreatic diseases and especially malignancies we therefore planned and conduct such a trial.

The PARC study is an open, randomized controlled trial investigating the survival of patients with primary non-metastatic locally advanced pancreatic cancer after trimodal therapy with gemcitabine-based chemoradiation and EGFR-targeting therapy with the monoclonal antibody cetuximab. The role and the mechanism of cetuximab in patient's chemoradiation regimen are evaluated. The toxicity, the disease-free interval and the quality of life are assessed. Different factors are tested for a potential role as predictive marker.

The results of the PARC trial will definitely advance clinical and scientific knowledge on the treatment of locally advanced pancreatic adenocarcinoma.

## Abbreviations

CRO Clinical Research Organisation

CRF Case Report Form

CTC Common Toxicity Criteria

CTV Clinical Target Volume

dkfz German Cancer Research Center

EBRT External Beam Radiation Therapy

EGFR Epidermal Growth Factor Receptor

GCP Good Clinical Practice

GTV Gross Target Volume

IMRT Intensity Modulated Radiation Therapy

IORT Intra-Operative Radiation Therapy

## Competing interests

The author(s) declare that they have no competing interests.

## Authors' contributions

The first two authors contributed equally. RK, MWM, SH, MWB and JD planned, coordinated and conducted the study. Medical care is covered by CT, BD, PB, KKH, and AA. RK, MWM, KKH and HF recruited patients and provided randomization. SN, CT, BD, PB, and AA took part in conducting the study. Scientific program is planned by RK and MWM and carried out by RK, MWM, PEH, AA and HF. SN provided technical assistance and quality control in radiation treatment planning and delivery. All authors read and approved the final manuscript.

## Pre-publication history

The pre-publication history for this paper can be accessed here:


